# Multi-Institutional Retrospective Analysis of Carbon-Ion Radiotherapy for Patients with Locally Advanced Adenocarcinoma of the Uterine Cervix

**DOI:** 10.3390/cancers13112713

**Published:** 2021-05-31

**Authors:** Noriyuki Okonogi, Ken Ando, Kazutoshi Murata, Masaru Wakatsuki, Shin-ei Noda, Daisuke Irie, Hiroshi Tsuji, Makio Shozu, Tatsuya Ohno

**Affiliations:** 1 National Institutes for Quantum and Radiological Science and Technology, QST Hospital, Chiba 263-8555, Japan; okonogi.noriyuki@qst.go.jp (N.O.); wakatsuki.masaru@qst.go.jp (M.W.); tsuji.hiroshi@qst.go.jp (H.T.); 2 Department of Radiation Oncology, Gunma University Graduate School of Medicine, Maebashi 371-8511, Japan; k.ando0906@gunma-u.ac.jp (K.A.); daisuke_i@gunma-u.ac.jp (D.I.); tohno@gunma-u.ac.jp (T.O.); 3 Department of Radiation Oncology, Saitama Medical University International Medical Center, Hidaka 350-1298, Japan; nodashin@saitama-med.ac.jp; 4 Department of Reproductive Medicine, Chiba University Graduate School of Medicine, Chiba 260-8677, Japan; shozu@faculty.chiba-u.jp

**Keywords:** carbon-ion radiotherapy, uterine cervical cancer, adenocarcinoma, long-term follow-up, cisplatin, concurrent chemoradiotherapy

## Abstract

**Simple Summary:**

We conducted a multi-institutional survey of carbon-ion radiotherapy (CIRT) for locally advanced adenocarcinoma (LAAC) of the uterine cervix. We retrospectively analyzed the clinical outcomes of patients with stage IIB–IVA LAAC of the uterine cervix who underwent chemo-CIRT or CIRT alone between April 2010 and April 2016. Fifty-five patients were enrolled in this study. The median follow-up period was 67.5 months. The 5-year overall survival (OS) and local control (LC) rates were 68.6% and 65.2%, respectively. Multivariate analysis showed that the initial tumor response within 6 months was significantly associated with LC and OS. The present study represents promising outcomes of CIRT or chemo-CIRT for LAAC of the uterine cervix, especially in the cases showing initial rapid regression of the tumor.

**Abstract:**

The clinical significance of carbon-ion radiotherapy (CIRT) for adenocarcinoma (AC) of the uterine cervix has been assessed in several single-institutional studies. To validate the significance, we conducted a multi-institutional survey of CIRT for locally advanced AC (LAAC) of the uterine cervix. We retrospectively analyzed the clinical outcomes of patients with stage IIB–IVA LAAC of the uterine cervix who underwent chemo-CIRT or CIRT alone between April 2010 and April 2016. Patients received 74.4 Gy (relative biological effectiveness [RBE]) in 20 fractions of CIRT or 55.2 Gy (RBE) in 16 fractions of CIRT plus three sessions of brachytherapy. Patients aged ≤ 70 years with adequate bone marrow and organ function were administered cisplatin weekly (40 mg/m^2^ per week for up to 5 weeks). Fifty-five patients were enrolled in this study. The median follow-up period was 67.5 months. The 5-year overall survival (OS) and local control (LC) rates were 68.6% and 65.2%, respectively. Multivariate analysis showed that the initial tumor response within 6 months was significantly associated with LC and OS. The present study represents promising outcomes of CIRT or chemo-CIRT for LAAC of the uterine cervix, especially in the cases showing initial rapid regression of the tumor.

## 1. Introduction

Uterine cervical cancer is the fourth most common type of cancer among women worldwide. In 2018, the global incidence of uterine cervical cancer was estimated to be 569,000, with 311,000 women dying from the disease [1]. Out of the different histological types of uterine cervical cancer, squamous cell carcinomas account for approximately 80% of all cervical cancers, and adenocarcinoma (AC) accounts for approximately 20% [2]. The incidence of adenocarcinoma of the uterine cervix has been on the rise [3,4].

Cisplatin-based concurrent chemoradiotherapy (CCRT) has been the standard treatment regimen for locally advanced cervical cancer as established by phase III randomized clinical trials and meta-analyses [5,6,7,8,9]. AC of the uterine cervix is known to be less radiosensitive than cervical squamous cell carcinoma. In fact, previous studies with radiation therapy (RT)/CCRT treatment showed lower local control (LC) rates for AC of the uterine cervix than cervical squamous cell carcinoma. The 5-year LC rates for patients who received CCRT for AC of the uterine cervix were 36–58% [10,11,12,13]. Since the use of image-guided brachytherapy (IGBT) has been accepted as a treatment for cervical cancer [14], several studies have reported improved LC rates [15,16]. Nevertheless, many recent reports indicate that patients with AC continue to have lower LC rates [17,18]. Therefore, new therapeutic strategies are required for AC of the uterine cervix.

Carbon-ion (C-ion) beams have improved dose-localization properties and have a high linear energy transfer in the Bragg peak, which is a biological advantage [19,20]. Due to these advantages, CIRT has been applied to various types of malignancies, including uterine cervical cancer [21]. We have investigated the clinical significance of CIRT for locally advanced AC (LAAC) of the uterine cervix for decades [22,23,24,25]. Wakatsuki et al. [22] reported that the 5-year LC rate for CIRT was 55%. Previously, we reported the feasibility of concurrent cisplatin use coupled with CIRT for uterine cervical cancer [24]. In that study, the concurrent administration of 40 mg/m^2^ of cisplatin coupled with CIRT was tolerable and showed favorable clinical outcomes. The 2-year LC and overall survival (OS) rates were 71% and 88%, respectively [24]. We recently reported the long-term significance of concurrent weekly cisplatin and CIRT (chemo-CIRT) for LAAC of the uterine cervix in a propensity score-matched analysis [25]. Thus, chemo-CIRT for LAAC is a promising therapeutic strategy.

However, these previous studies are single-institutional investigations. A multi-institutional investigation is warranted to validate the clinical significance of CIRT or chemo-CIRT for LAAC. Here, we report the results of a multi-institutional survey on CIRT or chemo-CIRT for LAAC of the uterine cervix.

## 2. Results

### 2.1. Patients and Tumor Characteristics

Fifty-five patients met our eligibility criteria. The median age of the patients was 55 years (range, 26–81 years). Thirty-seven patients had stage IIB, 15 had stage IIIB, and three had stage IVA disease. The median tumor size was 5.3 cm (range, 2.8–12.0 cm). Twenty-two patients had pelvic lymph node metastasis before the treatment. Forty-nine patients underwent CIRT regimen, while six patients underwent CIRT and brachytherapy regimen. Of the six patients who underwent CIRT and brachytherapy regimen, three received a combination of intracavitary and interstitial brachytherapy as brachytherapy treatment. Thirty-six patients received chemo-CIRT, while 19 patients received CIRT alone. Fifty patients had AC, and five patients had adenosquamous carcinoma. The patient and tumor characteristics are shown in Table 1.

### 2.2. Treatment Efficacy and Prognostic Factors

**Figure 1:**
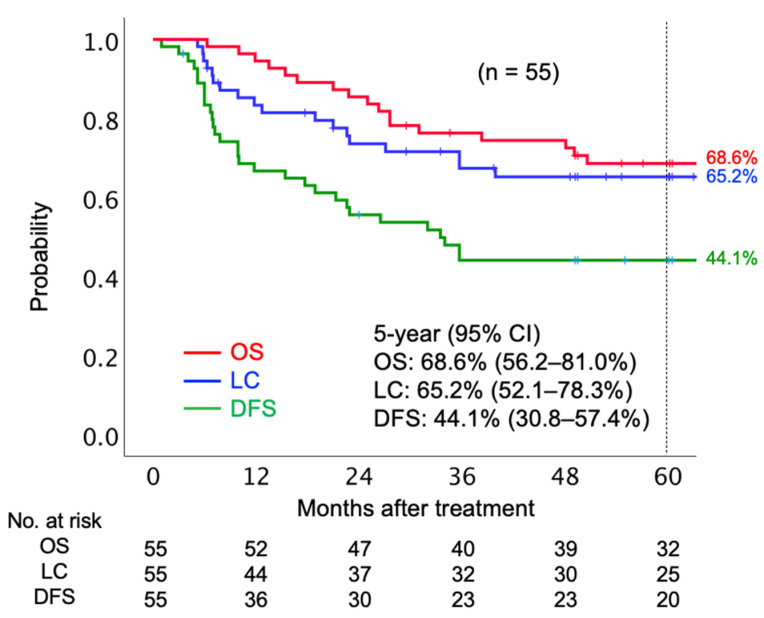
Kaplan–Meier curves of overall survival, local control, and disease-free survival rates for all patients analyzed. Number at risk is shown below the figure. Abbreviations: OS, overall survival; LC, local control; DFS, disease-free survival; CI, confidence interval.

The median follow-up period was 67.5 months. In terms of initial tumor response based on the Response Evaluation Criteria in Solid Tumors (RECIST) criteria, 41 (74.6%) patients achieved complete response (CR), 13 (23.6%) patients achieved partial response (PR), and one (1.8%) had stable disease. Twenty patients had local tumor recurrence before the final follow-up. Of 20 patients who had local recurrence, six patients underwent salvage surgery. Post-surgery, four patients were local-disease-free, and two patients showed re-recurrence in the local region by the final follow-up date. Twenty-one patients died before the final follow-up date, out of whom 18 died from the AC of the uterine cervix and three died due to other reasons (pulmonary thromboembolism, secondary malignancy, and old age). The 5-year OS, LC, and disease-free survival (DFS) rates were 68.6% (95% confidence interval [CI] 56.2–81.0%), 65.2% (95% CI 52.1–78.3%), and 44.1% (30.8–57.4%), respectively (Figure 1).

Table 2A shows the results of univariate analyses for prognostic factors in each clinical outcome. Median values were used to set the cutoff value for tumor size (5.3 cm). The International Federation of Gynecology and Obstetrics (FIGO 2008) staging was significantly associated with OS in the univariate analysis (5-year OS: IIB versus IIIB–IVA, 75.4% versus 54.3%, *p* = 0.019). Additionally, the initial tumor response was significantly associated with LC (5-year LC: CR versus non-CR, 76.8 versus 28.9, *p* < 0.001), DFS (5-year DFS: CR versus non-CR, 53.2% versus 15.5%, *p* = 0.001), and OS (5-year OS: CR versus non-CR, 82.6% versus 28.6%, *p* < 0.001). However, none of the other factors, including pelvic lymph node metastasis, tumor size, and concurrent use of cisplatin, were significantly correlated with the clinical outcomes. A multivariate analysis based on the Cox proportional-hazards model showed that the initial tumor response was significantly associated with LC (*p* = 0.003, hazard ratio [HR]: 0.227), DFS (*p* = 0.002, HR: 0.264), and OS (*p* = 0.002, HR: 0.253) (Table 2B).

### 2.3. Relationship between Clinical Outcomes and Histological Subtypes

**Figure 2:**
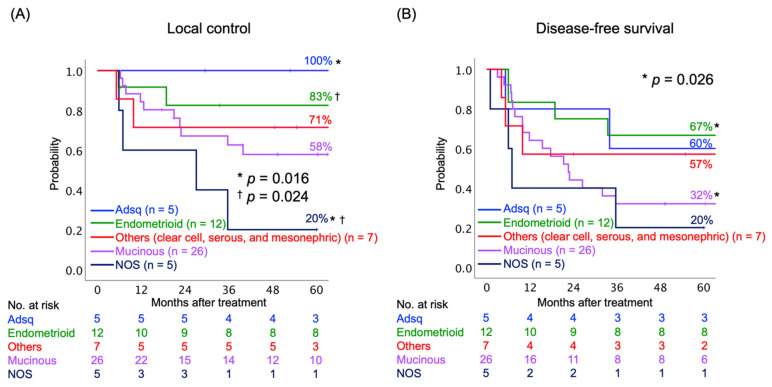
Kaplan–Meier curves of (**A**) local control and (**B**) disease-free survival rates classified by histological subtypes. Number at risk is shown below figures. Abbreviations: Adsq, adenosquamous; NOS, not otherwise specified. * ^†^ indicate the corresponding pairs.

Next, we assessed the LC and DFS rates according to the histological subtypes of AC. The histological subtypes were divided into five groups: (i) adenosquamous carcinoma (Adsq); (ii) endometrioid AC; (iii) mucinous AC, including usual type, gastric type, and signet-ring cell type; (iv) other subtypes, including clear cell, serous, and mesonephric AC; and (v) AC not otherwise specified (NOS). The AC NOS group consisted mainly of poorly differentiated tumors. The 5-year LC rates were statistically different between Adsq and AC NOS (100% and 20%, respectively, *p* = 0.016), as well between endometrioid AC and AC NOS (80% and 20%, respectively, *p* = 0.024) (Figure 2A). The 5-year DFS rates were statistically different between endometrioid and mucinous AC (67% and 32%, respectively, *p* = 0.016) (Figure 2B).

### 2.4. Acute and Late Toxicities

The acute and late toxicities observed in patients are listed in Table 3A–C. In terms of acute toxicity among patients, three patients developed grade 3 neutrophil decrease and one patient developed grade 3 hemoglobin decrease. Only one patient developed grade 3 nausea, which required tentative transvenous hydration. Thus, only five developed grade 3 toxicity. None of the patients developed grade 4 toxicity. No statistically significant differences were observed in the incidence of acute toxicity between the CIRT and chemo-CIRT groups (Table 3A,B).

Only one patient developed grade 3 rectum/sigmoid toxicity in the CIRT alone group in terms of late toxicity. Meanwhile, three patients developed grade 3 or worse rectum/sigmoid toxicity, two patients developed grade 3 genitourinary toxicity, and one patient developed both grade 3 rectum/sigmoid and genitourinary toxicities in the chemo-CIRT group. Thus, six patients developed grade 3 or worse late toxicity in the chemo-CIRT group. Of six patients who developed grade 3 or worse late toxicity in the chemo-CIRT group, three had undergone salvage surgery. No statistically significant differences were observed in the incidence of late toxicity between the CIRT and chemo-CIRT groups (Table 3C). There was no statistically significant difference in the incidence of acute or late severe toxicities between the radiation regimens.

## 3. Discussion

This study is the first multi-institutional study to assess the long-term outcomes of CIRT or chemo-CIRT for LAAC. A recent systematic review showed that CIRT could be considered a safe, effective, and feasible therapy for gynecological carcinomas [26]. However, all of the previous studies on CIRT for gynecological carcinomas, including AC of the uterine cervix, were single-institutional surveys. Previously conducted phase I/II studies assessing the feasibility of CIRT for LAAC showed that 5-year LC and OS rates were 55% and 38%, respectively [22]. A recent phase I/II study assessing the feasibility of concurrent cisplatin administration with CIRT for LAAC showed that 2-year LC and OS rates were 71% and 88%, respectively [23]. The present study on CIRT or chemo-CIRT for LAAC shows 5-year LC and OS rates of 65% and 69%, respectively. Currently, CCRT including brachytherapy is the standard of care for cervical cancer. As shown in Table 4, however, preceding studies of conventional RT or CCRT reported 5-year OS rates of up to 33% for LAAC of the uterine cervix [10,11,12,13,27,28,29]. Some of these studies consisted of CCRT with IGBT including interstitial brachytherapy; nevertheless, the clinical outcomes have not been improved [27,28]. Thus, CIRT or chemo-CIRT seems to be another promising strategy for LAAC of the uterine cervix.

We previously demonstrated that chemo-CIRT for LAAC of the uterine cervix is associated with a long-term survival benefit using a propensity score-matched analysis, especially in stage IIIB disease [25]. In terms of prognostic factors based on the clinical outcomes of the present study, concurrent uses of chemotherapy did not affect LC, DSF, or OS rate. This discrepancy may be explained by the selection criteria or bias for the patients. The patients who received chemo-CIRT had adequate organ and bone marrow function and were ≤70 years of age. As demonstrated in the current study [25], patients with LAAC of the uterine cervix should be treated with chemo-CIRT if patients have adequate organ and bone marrow functions. However, even if the patient was not administered concurrent chemotherapy, CIRT alone showed comparable and favorable results compared to chemo-CIRT for LAAC of the uterine cervix. This finding encourages the use of CIRT for patients who do not have adequate organs and bone marrow function or for elderly patients. It is worth noting that the initial tumor response at 6 months post-treatment was significantly associated with LC, DFS, and OS in the present study. In the case of definitive RT being performed for uterine cervical cancer, very few reports have investigated the prognostic factors specific to AC of the uterine cervix. Yokoi et al. [27] demonstrated that a large tumor size and incomplete response to RT were independent prognostic factors for DFS in patients with AC of the uterine cervix when definitive RT was performed. The fact that tumor size was not associated with any clinical outcomes in this study may have been the result of the inherent biological advantage of CIRT. Considering the slow shrinkage of AC of the uterine cervix post-treatment, careful monitoring of up to 6 months after CIRT is needed.

Recent propensity scores matching analyses and a population-based analysis demonstrated that patients with AC or Adsq had poor survival outcomes than those with squamous cell carcinoma [27,29,30]. In addition, several studies have suggested that AC is different from squamous cell carcinoma based on its molecular pathogenesis [31,32]. However, there are very few studies on whether the subtypes of AC of the uterine cervix affects prognosis. In the present study, Adsq or endometrioid AC showed a favorable prognosis compared to mucinous AC or AC NOS (consisting mainly of poorly differentiated tumors). The molecular biological differences between the histological subtypes of AC of the uterine cervix have not been elucidated yet. Further studies with a larger number of patients are needed to determine whether the prognosis varies for each histological subtype. Understanding the biological differences between the histological subtypes may lead to an appropriate treatment strategy for AC of the uterine cervix in the future.

In terms of acute toxicity, only four patients developed grade 3 or worse toxicities in all 55 patients in the present study. Out of 36 patients who received chemo-CIRT, only two (6%) developed grade 3 hematological toxicities. In a systematic review, Kirwan et al. [33] reported that the incidence of acute hematological toxicities of grade ≥ 3 was up to 27.6% for conventional CCRT. The lower incidence of acute toxicity in our study may be explained by the excellent dose distribution of CIRT, which reduces the dose to the bone marrow in the pelvic region. In terms of late toxicity, only one out of 19 patients developed grade 3 toxicity in the CIRT group. Meanwhile, six out of 36 patients developed grade 3 or worse toxicity in the chemo-CIRT group. No statistically significant differences were observed in the incidence of late toxicity between the CIRT and chemo-CIRT groups. In fact, three of six patients who developed grade 3 or worse late toxicity in the chemo-CIRT group underwent salvage surgery. Thus, the concurrent use of cisplatin with CIRT does not exacerbate the late toxicities of CIRT. The use of salvage surgery is carefully considered for the central recurrence of the uterine cervix cancer when prior conventional RT is performed [2]. Indeed, of the 20 patients who had local recurrence, six patients underwent salvage surgery, of whom four patients were local-disease-free by the final follow-up date in our study. Although deep consideration for late toxicity is needed, salvage surgery may be a treatment option for central-recurrent cases when CIRT as well as conventional RT are applied.

The present study included several limitations such as the retrospective analysis, various treatment regimens, and time-based differences in patient care. Although all patients included in the present study were informed that the standard care for LAAC of the uterine cervix is CCRT including X-ray and brachytherapy, selection bias cannot be excluded. In addition, as CIRT is not a commonly used technique at present, the generalizability of these findings may be limited. Since 2016, we conducted a nationwide prospective registry including patient follow-up data after CIRT or chemo-CIRT for patients with LAAC. It is important to confirm whether the oncologic outcomes obtained from this patient cohort can validate the results of our present study. The current standard of care for LAAC of the uterine cervix is CCRT including X-ray and brachytherapy. Thus, if necessary, a randomized clinical trial to clarify the difference in clinical outcomes between CIRT and standard therapy would be warranted in future.

## 4. Materials and Methods

### 4.1. Eligibility

We explained to all candidate patients that the standard care for locally advanced cervical cancer is CCRT including X-ray and brachytherapy. These patients had been fully informed of the expected benefits and possible toxicities associated with CIRT were treated with CIRT, then only patients who had provided written consent were treated with CIRT. In addition, before undergoing CIRT, all patients were evaluated for CIRT eligibility by the cancer board, which consisted of gynecologic oncologists and radiation oncologists with sufficient clinical experience.

Patients who received CIRT for LAAC between April 2010 and April 2016 and who met the following eligibility criteria were enrolled: (i) histologically proven AC of the uterine cervix, (ii) FIGO stage IIB to IVA, (iii) abdominal computed tomography (CT) did not reveal lymph nodes > 1 cm in diameter in the abdominal para-aortic region; (iv) no prior treatment for cervical cancer; and (v) estimated survival period of at least 6 months. In addition, patients who received chemo-CIRT were ≤70 years of age and with adequate bone marrow (hemoglobin level, 10.0 g/dL; leukocyte count, 3000/mL; and platelet count, 100,000/mL), renal, and hepatic (serum creatinine level, <1.5 mg/dL; total bilirubin level, <1.5 mg/mL; aspartate/alanine aminotransferase level, <100 IU/dL) function. The exclusion criteria for this study were (i) unmanageable critical complications, (ii) active double cancer, (iii) rectal invasion of the tumor, and (iv) a history of pelvic and abdominal RT or chemotherapy.

All patients underwent contrast-enhanced chest-abdomen-pelvis computed tomography (CT), pelvic magnetic resonance imaging (MRI), and 18F-fluorodeoxyglucose positron emission tomography-CT scans for oncologic staging before beginning treatment. Tumor size was assessed by pelvic examination and MRI.

### 4.2. Carbon-Ion Radiotherapy

The treatment procedures are described in detail in previous reports [22,23,24,25]. All patients underwent CT in the supine position using customized cradles and were immobilized with a low-temperature thermoplastic sheet. A set of 2.0- or 2.5-mm-thick CT images was used for treatment planning and dose calculation, and the evaluations were performed using XiO-N (Elekta, Stockholm, Sweden) or XiO-N2 software (National Institute of Radiological Sciences, Chiba, Japan). The radiation dose was calculated for the target volume and normal surrounding structures and was expressed in Gy (relative biological effectiveness [RBE]), which was defined as the physical dose multiplied by the RBE of the C-ions using the Kanai model [20,34].

CIRT for LAAC of the uterine cervix consisted of whole pelvic irradiation and local boost [22,23,24,25]. Details of the target definitions are described in Table S1. Whole-pelvic irradiation (PTV (planned target volume) 1), irradiation of the uterus with tumor infiltrating region and swollen pelvic lymph nodes (PTV2), and local tumor (PTV3) boost irradiation were performed by CIRT. The irradiation for PTV3 was substituted with three sessions of 3D-IGBT. CIRT was administered once daily for 4 days a week (Tuesday to Friday). The 3D-IGBT for PTV3 was administered in three sessions in 2 weeks. The two different radiation regimens in the present study were determined according to the treatment policies of the participating centers. Both methods are recognized as CIRT methods by the responsible ministry and relevant radiotherapy society [35].

At each treatment session of CIRT, the patient was positioned using an orthogonal digital X-ray positioning system. The patients received laxatives to prevent constipation during the treatment period. To minimize internal motion, 100–150 mL of normal saline was injected into the bladder. During the CIRT for PTV2 or PTV3, vaginal packing (i.e., cotton pads soaked in contrast medium) or vaginal devices [36] were used to allow visualization of the surface of the cervix by X-ray.

The CIRT dose was prescribed at the isocenter of the PTVs. A standard regimen of CIRT without brachytherapy included a total dose of 36.0 Gy to the cervical tumor in 12 fractions for the PTV1, 19.2 Gy (RBE) in 4 fractions for the PTV2, and 19.2 Gy (RBE) in 4 fractions for PTV3. The IGBT dose for PTV3D90 (minimum dose delivered to 90% of the PTV3) was set as 6 Gy per fraction. The treatment outline and dose fractionation schedule are shown in Figure S1.

### 4.3. Chemotherapy

A weekly cisplatin dose of 40 mg/m^2^ was administered during the treatment period for up to five courses. Chemotherapy was discontinued if (1) the patient developed hematological toxicity ≥ grade 3, (2) serum creatinine levels were ≥1.5 mg/dL, or (3) aspartate and alanine aminotransferase levels were ≥100 IU/dL. Chemotherapy was also discontinued if the patient developed ≥ grade 3 complications in the GI tract or urinary system.

### 4.4. Evaluation and Statistical Analyses

The histological subtype was evaluated by pathologists according to the WHO histological classification of tumors of the uterine cervix in 2014 [37]. Acute toxicities were classified according to the National Cancer Institute Common Terminology Criteria for Adverse Events, Version 4.0, with a maximum reaction within 3 months after initiation of therapy. Late toxicities were classified according to the Radiation Therapy Oncology Group/European Organization for Research and Treatment of Cancer scoring system [38]. 

Tumor response was assessed six months post-treatment in accordance with the RECIST v1.1 [39]. LC, OS, and DFS rates were calculated using the Kaplan–Meier method. The log-rank test was used for the univariate analyses. All clinical factors analyzed in the univariate analyses were included in the multivariate analysis, and a Cox proportional-hazards regression model was used. Chi-square tests were used to assess the incidence of severe toxicities. All statistical analyses were performed using Statistical Package for the Social Sciences for Macintosh, version 27.0 (IBM Inc., Armonk, NY, USA). A two-sided p < 0.05 was considered statistically significant in all tests.

## 5. Conclusions

We reported the results of a multi-institutional survey on CIRT or chemo-CIRT for LAAC of the uterine cervix. Considering the favorable outcomes of the present study, CIRT or chemo-CIRT for LAAC of the uterine cervix may be considered a new treatment strategy for this disease.

## Figures and Tables

**Table 1:** Patients and tumor characteristics (*n* = 55).

Characteristics	*n* = 55
Age (median), years	26–81 (55)
Follow-up period (median), months	6.3–109.6 (67.5)
FIGO stage (2008)	
IIB	37
IIIB	15
IVA	3
Histological subtypes	
Endocervical adenocarcinoma, usual type	14
Mucinous adenocarcinoma	10
Mucinous carcinoma, gastric type	1
Mucinous carcinoma, signet-ring cell type	1
Endometrioid adenocarcinoma	12
Clear cell adenocarcinoma	3
Serous adenocarcinoma	3
Mesonephric adenocarcinoma	1
Adenocarcinoma, NOS	5
Adenosquamous carcinoma	5
Pelvic LN metastasis	
No	33
Yes	22
Tumor size (median), cm	2.8–12.0 (5.3)
Weekly CDDP administrations	
No	19
Yes	36
1 course	1
2 courses	0
3 courses	3
4 courses	6
5 courses	26

FIGO = International Federation of Gynecology and Obstetrics; NOS = not otherwise specified; LN = lymph node; CDDP = cisplatin.

**Table 2:** Assessments of prognostic factors with (**A**) univariate analysis and (**B**) multivariate analysis.

**(A)**							
**Factor**	**No. of Patients**	**LC**		**DFS**		**OS**	
		**5-Year (%)**	***p* Value**	**5-Year (%)**	***p* Value**	**5-Year (%)**	***p* Value**
FIGO stage (2008)			0.243		0.684		0.019
IIB	37	71.8		48.6		75.4	
IIIB-IVA	18	50.8		33.2		54.3	
Pelvic LN metastasis			0.221		0.274		0.572
No	33	60.5		48.9		72.2	
Yes	22	73.5		40.5		63.3	
Tumor size			0.896		0.392		0.741
≤5.3 cm	27	68.9		38.3		67.6	
>5.3 cm	28	61.6		50.2		70.2	
Concurrent CDDP			0.827		0.805		0.102
No	19	65.4		46.3		57.4	
Yes	36	65.4		42.9		71.2	
Initial tumor response			<0.001		0.001		<0.001
CR	41	76.8		53.2		82.6	
Non-CR	15	28.9		15.5		28.6	
**(B)**							
**Factor**		**LC**		**DFS**		**OS**	
		***p* Value**	**HR (95% CI)**	***p* Value**	**HR (95% CI)**	***p* Value**	**HR (95% CI)**
FIGO stage (2008)		0.672	-	0.767	-	0.075	-
Pelvic LN metastasis		0.286	-	0.664	-	0.611	-
Tumor size (≤5.3 cm)		0.972	-	0.261	-	0.336	-
Concurrent CDDP		0.838	-	0.826	-	0.181	-
Initial tumor response		0.003	0.227 (0.086–0.598)	0.002	0.264 (0.115–0.610)	0.002	0.253 (0.104–0.615)

LC = local control; DFS = disease-free survival; OS = overall survival; FIGO = International Federation of Gynecology and Obstetrics; LN = lymph node; CDDP = cisplatin; CR = complete response; HR = hazard ratio.

**Table 3:** Lists of (**A**) acute hematological, (**B**) acute non-hematological, and (**C**) late non-hematological toxicities.

**(A)**							
**Treatment** **Regimen**	**Number of Patients**	**Neutrophil Decrease** **Grade**		**Hemoglobin Decrease** **Grade**		**Platelet Decrease** **Grade**	
		**0–2**	**3–4**	**0–2**	**3–4**	**0–2**	**3–4**
CIRT alone	19	18	1	18	1	19	0
Chemo-CIRT	36	34	2	36	0	36	0
*p*-value		0.929		0.168		N/A	
**(B)**							
**Treatment** **Regimen**	**Number of Patients**	**Nausea/Vomiting** **Grade**		**Lower Gastrointestinal** **Grade**		**Genitourinary** **Grade**	
		**0–2**	**3–4**	**0–2**	**3–4**	**0–2**	**3–4**
CIRT alone	19	19	0	19	0	19	0
Chemo-CIRT	36	35	1	36	0	36	0
*p*-value		0.463		N/A		N/A	
**(C)**							
**Treatment** **Regimen**	**Number of Patients**	**Rectum/Sigmoid** **Grade**		**Small Intestine** **Grade**		**Genitourinary** **Grade**	
		**0–2**	**3–4**	**0–2**	**3–4**	**0–2**	**3–4**
CIRT alone	19	18	1	19	0	19	0
Chemo-CIRT	36	32	4 *	36	0	33	3 ^†^
*p*-value		0.473		N/A		0.195	

CIRT = carbon-ion radiotherapy; N/A = not available. * One patient required colostomy when salvage operation was performed, another patient required colostomy due to local tumor recurrence, and the other patient required colostomy due to radiation proctitis. ^†^ Two patients required urinary diversion surgery when salvage operation was performed; the other patient had T4 disease (bladder invasion) and developed vesicovaginal fistula after treatment.

**Table 4:** Review of previous literature and this study.

Author (Year), References	Stage	Number	Treatment	5y LC	5y OS
				(%)	(%)
Eifel PJ (1990) [10]	III–IV	48	RT	52%	31%
Farley JH (2003) [11]	III	13	RT/CCRT	N/R	32%
Niibe Y (2010) [12]	IIIB	61	RT/CCRT	36%	20%
Huang YT (2011) [13]	III	38	RT/CCRT	58%	N/R
	IVA	3		0%	
Yokoi E (2017) [27]	IIB–IVA	24	CCRT (IGBT)	N/R	27%
Miyasaka Y (2020) [28]	III–IVA	35	RT/CCRT (IGBT)	62% (IB–IVA)	26%
Zhang J (2020) [29]	II	149	CCRT	N/R	33%
	III	65			33%
	IVA	49			9%
Present study (2021)	IIB–IVA (Good responders *)	55	CIRT/ chemo-CIRT	65%	69%
		41		(77%)	(83%)

RT = radiotherapy; CCRT = concurrent chemoradiotherapy; N/R = not reported; IGBT = image-guided brachytherapy; CIRT = carbon-ion radiotherapy; Chemo-CIRT = chemo-carbon-ion radiotherapy. * Good responders indicate patients who achieved CR at 6 months post-treatment in accordance with the RECIST v1.1.

## Data Availability

The data presented in this study are available on request from the corresponding author. The data are not publicly available due to restrictions of patients’ privacy or ethical aspect.

## Supplementary Materials

The following are available online at https://www.mdpi.com/article/10.3390/cancers13112713/s1, Figure S1: Outline of treatment of the present study, Table S1: Details of target definitions.

## References

[1] Mathew A., George P.S. (2009). Trends in Incidence and Mortality Rates of Squamous Cell Carcinoma and Adenocarcinoma of Cervix--Worldwide. Asian Pac. J. Cancer Prev..

[2] NCCN Guidelines version 1.2021 Cervical Cancer. https://www.nccn.org/professionals/physician_gls/pdf/cervical_blocks.pdf.

[3] Wang S.S., Sherman M.E., Hildesheim A., Lacey J.V., Devesa S. (2004). Cervical Adenocarcinoma and Squamous Cell Carcinoma Incidence Trends Among White Women and Black Women in the United States for 1976–2000. Cancer.

[4] Bray F., Carstensen B., Møller H., Zappa M., Zakelj M.P., Lawrence G., Hakama M., Weiderpass E. (2005). Incidence Trends of Adenocarcinoma of the Cervix in 13 European Countries. Cancer Epidemiol. Biomarkers Prev..

[5] Rose P.G., Bundy B.N., Watkins E.B., Thigpen J.T., Deppe G., Maiman M.A., Clarke-Pearson D.L., Insalaco S. (1999). Concurrent Cisplatin-Based Radiotherapy and Chemotherapy for Locally Advanced Cervical Cancer. N. Engl. J. Med..

[6] Green J.A., Kirwan J.M., Tierney J.F., Symonds P., Fresco L., Collingwood M., Williams C.J. (2001). Survival and Recurrence After Concomitant Chemotherapy and Radiotherapy for Cancer of the Uterine Cervix: A Systematic Review and Meta-Analysis. Lancet.

[7] Eifel P.J., Winter K., Morris M., Levenback C., Grigsby P.W., Cooper J., Rotman M., Gershenson D., Mutch D.G. (2004). Pelvic Irradiation with Concurrent Chemotherapy Versus Pelvic and Para-Aortic Irradiation for High-Risk Cervical Cancer: An Update of Radiation Therapy Oncology Group Trial (RTOG) 90-01. J. Clin. Oncol..

[8] Vale C., Tierney J., Stewart L. (2006). Concomitant Chemoradiotherapy for Cervical Cancer: A Systematic Review and Meta-Analysis of Individual Patient Data. Gynecol. Oncol..

[9] Chemoradiotherapy for Cervical Cancer Meta-Analysis Collaboration (2008). Reducing Uncertainties About the Effects of Chemoradiotherapy for Cervical Cancer: A Systematic Review and Meta-Analysis of Individual Patient Data From 18 Randomized Trials. J. Clin. Oncol..

[10] Eifel P.J., Morris M., Oswald M.J., Wharton J.T., Delclos L. (1990). Adenocarcinoma of the Uterine Cervix. Prognosis and Patterns of Failure in 367 Cases. Cancer.

[11] Farley J.H., Hickey K.W., Carlson J.W., Rose G.S., Kost E.R., Harrison T.A. (2003). Adenosquamous Histology Predicts a Poor Outcome for Patients with Advanced-Stage, but Not Early-Stage, Cervical Carcinoma. Cancer.

[12] Niibe Y., Kenjo M., Onishi H., Ogawa Y., Kazumoto T., Ogino I., Tsujino K., Harima Y., Takahashi T., Anbai A. (2010). High-Dose-Rate Intracavitary Brachytherapy Combined with External Beam Radiotherapy for stage IIIb Adenocarcinoma of the Uterine Cervix in Japan: A Multi-Institutional Study of Japanese Society of Therapeutic Radiology and Oncology 2006–2007 (Study of JASTRO 2006–2007). Jpn. J. Clin. Oncol..

[13] Huang Y.T., Wang C.C., Tsai C.S., Lai C.H., Chang T.C., Chou H.H., Hsueh S., Chen C.K., Lee S.P., Hong J.H. (2011). Long-Term Outcome and Prognostic Factors for Adenocarcinoma/Adenosquamous Carcinoma of Cervix After Definitive Radiotherapy. Int. J. Radiat. Oncol. Biol. Phys..

[14] Pötter R., Haie-Meder C., Van Limbergen E., Barillot I., De Brabandere M., Dimopoulos J., Dumas I., Erickson B., Lang S., Nulens A. (2006). Recommendations from Gynaecological (GYN) GEC ESTRO Working Group (II): Concepts and Terms in 3D Image-Based Treatment Planning in Cervix Cancer brachytherapy-3D Dose Volume Parameters and Aspects of 3D Image-Based Anatomy, Radiation Physics, Radiobiology. Radiother. Oncol..

[15] Pötter R., Georg P., Dimopoulos J.C., Grimm M., Berger D., Nesvacil N., Georg D., Schmid M.P., Reinthaller A., Sturdza A. (2011). Clinical Outcome of Protocol Based Image (MRI) Guided Adaptive Brachytherapy Combined With 3D Conformal Radiotherapy with or Without Chemotherapy in Patients with Locally Advanced Cervical Cancer. Radiother. Oncol..

[16] Sturdza A., Pötter R., Fokdal L.U., Haie-Meder C., Tan L.T., Mazeron R., Petric P., Šegedin B., Jurgenliemk-Schulz I.M., Nomden C. (2016). Image Guided Brachytherapy in Locally Advanced Cervical Cancer: Improved Pelvic Control and Survival in RetroEMBRACE, a Multicenter Cohort Study. Radiother. Oncol..

[17] Minkoff D., Gill B.S., Kang J., Beriwal S. (2015). Cervical Cancer Outcome Prediction to High-Dose Rate Brachytherapy Using Quantitative Magnetic Resonance Imaging Analysis of Tumor Response to External Beam Radiotherapy. Radiother. Oncol..

[18] Kusada T., Toita T., Ariga T., Maemoto H., Hashimoto S., Shiina H., Kakinohana Y., Heianna J., Nagai Y., Kudaka W. (2018). Computed Tomography-Based Image-Guided Brachytherapy for Cervical Cancer: Correlations Between Dose-Volume Parameters and Clinical Outcomes. J. Radiat. Res..

[19] Kanai T., Furusawa Y., Fukutsu K., Itsukaichi H., Eguchi-Kasai K., Ohara H. (1997). Irradiation of Mixed Beam and Design of Spread-Out Bragg Peak for Heavy-Ion Radiotherapy. Radiat. Res..

[20] Kanai T., Endo M., Minohara S., Miyahara N., Koyama-ito H., Tomura H., Matsufuji N., Futami Y., Fukumura A., Hiraoka T. (1999). Biophysical Characteristics of HIMAC Clinical Irradiation System for Heavy-Ion Radiation Therapy. Int. J. Radiat. Oncol. Biol. Phys..

[21] Kamada T., Tsujii H., Blakely E.A., Debus J., De Neve W., Durante M., Jäkel O., Mayer R., Orecchia R., Pötter R. (2015). Carbon Ion Radiotherapy in Japan: An Assessment of 20 Years of Clinical Experience. Lancet Oncol..

[22] Wakatsuki M., Kato S., Ohno T., Karasawa K., Kiyohara H., Tamaki T., Ando K., Tsujii H., Nakano T., Kamada T. (2014). Clinical Outcomes of Carbon Ion Radiotherapy for Locally Advanced Adenocarcinoma of the Uterine Cervix in phase 1/2 Clinical Trial (Protocol 9704). Cancer.

[23] Ohno T., Noda S.E., Murata K., Yoshimoto Y., Okonogi N., Ando K., Tamaki T., Kato S., Hirakawa T., Kanuma T. (2018). Phase I Study of Carbon Ion Radiotherapy and Image-Guided Brachytherapy for Locally Advanced Cervical Cancer. Cancers.

[24] Okonogi N., Wakatsuki M., Kato S., Karasawa K., Kiyohara H., Shiba S., Kobayashi D., Nakano T., Kamada T., Shozu M. (2018). Clinical Outcomes of Carbon Ion Radiotherapy with Concurrent Chemotherapy for Locally Advanced Uterine Cervical Adenocarcinoma in a phase 1/2 Clinical Trial (Protocol 1001). Cancer Med..

[25] Okonogi N., Wakatsuki M., Kato S., Karasawa K., Miyasaka Y., Murata H., Nakano T., Kamada T., Shozu M., Working Group of Gynecological Tumors (2019). A Phase 1/2 Study of Carbon Ion Radiation Therapy with Concurrent Chemotherapy for Locally Advanced Uterine Cervical Squamous Cell Carcinoma (Protocol 1302). Int. J. Radiat. Oncol. Biol. Phys..

[26] Wang L., Wang X., Zhang Q., Ran J., Geng Y., Feng S., Li C., Zhao X. (2019). Is There a Role for Carbon Therapy in the Treatment of Gynecological Carcinomas? A Systematic Review. Future Oncol..

[27] Yokoi E., Mabuchi S., Takahashi R., Matsumoto Y., Kuroda H., Kozasa K., Kimura T. (2017). Impact of Histological Subtype on Survival in Patients with Locally Advanced Cervical Cancer That Were Treated With Definitive Radiotherapy: Adenocarcinoma/Adenosquamous Carcinoma Versus Squamous Cell Carcinoma. J. Gynecol. Oncol..

[28] Miyasaka Y., Yoshimoto Y., Murata K., Noda S.E., Ando K., Ebara T., Okonogi N., Kaminuma T., Yamada S., Ikota H. (2020). Treatment Outcomes of Patients with Adenocarcinoma of the Uterine Cervix After Definitive Radiotherapy and the Prognostic Impact of Tumor-Infiltrating CD8+ Lymphocytes in Pre-Treatment Biopsy Specimens: A Multi-Institutional Retrospective Study. J. Radiat. Res..

[29] Zhang J., Qin L., Chen H.M., Hsu H.C., Chuang C.C., Chen D., Wu S.Y. (2020). Overall Survival, Locoregional Recurrence, and Distant Metastasis of Definitive Concurrent Chemoradiotherapy for Cervical Squamous Cell Carcinoma and Adenocarcinoma: Before and After Propensity Score Matching Analysis of a Cohort Study. Am. J. Cancer Res..

[30] Zhou J., Wu S.G., Sun J.Y., Li F.Y., Lin H.X., Chen Q.H., He Z.Y. (2017). Comparison of Clinical Outcomes of Squamous Cell Carcinoma, Adenocarcinoma, and Adenosquamous Carcinoma of the Uterine Cervix After Definitive Radiotherapy: A Population-Based Analysis. J. Cancer Res. Clin. Oncol..

[31] Wright A.A., Howitt B.E., Myers A.P., Dahlberg S.E., Palescandolo E., Van Hummelen P., MacConaill L.E., Shoni M., Wagle N., Jones R.T. (2013). Oncogenic Mutations in Cervical Cancer: Genomic Differences Between Adenocarcinomas and Squamous Cell Carcinomas of the Cervix. Cancer.

[32] Ojesina A.I., Lichtenstein L., Freeman S.S., Pedamallu C.S., Imaz-Rosshandler I., Pugh T.J., Cherniack A.D., Ambrogio L., Cibulskis K., Bertelsen B. (2014). Landscape of Genomic Alterations in Cervical Carcinomas. Nature.

[33] Kirwan J.M., Symonds P., Green J.A., Tierney J., Collingwood M., Williams C.J. (2003). A Systematic Review of Acute and Late Toxicity of Concomitant Chemoradiation for Cervical Cancer. Radiother. Oncol..

[34] Inaniwa T., Kanematsu N., Matsufuji N., Kanai T., Shirai T., Noda K., Tsuji H., Kamada T., Tsujii H. (2015). Reformulation of a Clinical-Dose System for Carbon-Ion Radiotherapy Treatment Planning at the National Institute of Radiological Sciences, Japan. Phys. Med. Biol..

[35] Authorized Treatment Policy of Carbon-Ion Radiotherapy for Locally Advanced Cervical Cancer, Japanese Society for Radiation Oncology (Written in Japanese). https://www.jastro.or.jp/medicalpersonnel/particle_beam/pdf/2018/S-J-5-1.pdf.

[36] Kubota Y., Ohno T., Kawashima M., Murata K., Okonogi N., Noda S.E., Tsuda K., Sakai M., Tashiro M., Nakano T. (2019). Development of a Vaginal Immobilization Device: A Treatment-Planning Study of Carbon-Ion Radiotherapy and Intensity-Modulated Radiation Therapy for Uterine Cervical Cancer. Anticancer Res..

[37] Histopathology of the Uterine Cervix, WHO 2014. https://screening.iarc.fr/atlasclassifwho.php.

[38] Cox J.D., Stetz J., Pajak T.F. (1995). Toxicity Criteria of the Radiation Therapy Oncology Group (RTOG) and the European Organization for Research and Treatment of Cancer (EORTC). Int. J. Radiat. Oncol. Biol. Phys..

[39] Eisenhauer E.A., Therasse P., Bogaerts J., Schwartz L.H., Sargent D., Ford R., Dancey J., Arbuck S., Gwyther S., Mooney M. (2009). New Response Evaluation Criteria in Solid Tumours: Revised RECIST Guideline (Version 1.1). Eur. J. Cancer.

