# Supplementary material for: Multi-Institutional Retrospective Analysis of Carbon-Ion Radiotherapy for Patients with Locally Advanced Adenocarcinoma of the Uterine Cervix
Source: Cancers (Basel). 2021 May 31;13(11):2713. doi: 10.3390/cancers13112713 (PMC8198465; doi:10.3390/cancers13112713)
Supplement: Supplementary file 1 [file cancers-13-02713-s001.zip › cancers-1250417-supplementary.pdf]

cancers

MDPI

Article

Multi-Institutional Retrospective Analysis of Carbon-Ion Radiotherapy for Patients with Locally Advanced Adenocarcinoma of the Uterine Cervix

Noriyuki Okonogi [1], Ken Ando [2,†], Kazutoshi Murata [1,*,†], Masaru Wakatsuki [1], Shin-ei Noda [3], Daisuke Irie [2], Hiroshi Tsuji [1], Makio Shozu [4] and Tatsuya Ohno [2]

[1] National Institutes for Quantum and Radiological Science and Technology, QST Hospital, Chiba 263-8555, Japan; okonogi.noriyuki@qst.go.jp (N.O.); wakatsuki.masaru@qst.go.jp (M.W.); tsuji.hiroshi@qst.go.jp (H.T.)
[2] Department of Radiation Oncology, Gunma University Graduate School of Medicine, Maebashi 371-8511, Japan; k.ando0906@gunma-u.ac.jp (K.A.); daisuke_i@gunma-u.ac.jp (D.I.); tohno@gunma-u.ac.jp (T.O.)
[3] Department of Radiation Oncology, Saitama Medical University International Medical Center, Hidaka 350-1298, Japan; nodashin@saitama-med.ac.jp
[4] Department of Reproductive Medicine, Chiba University Graduate School of Medicine, Chiba 260-8677, Japan; shozu@faculty.chiba-u.jp
* Correspondence: murata.kazutoshi@qst.go.jp; Tel.: +81-43-206-3306; Fax: +81-43-256-6506
† These authors contributed to this study equally.

Citation: Okonogi, N.; Ando, K.; Murata, K.; Wakatsuki, M.; Noda, S.-e.; Irie, D.; Tsuji, H.; Shozu, M.; Ohno, T. Multi-Institutional Retrospective Analysis of Carbon-Ion Radiotherapy for Patients with Locally Advanced Adenocarcinoma of the Uterine Cervix. *Cancers* **2021**, *13*, 2713. https://doi.org/10.3390/cancers13112713

Academic Editor: Arya Amini

Publisher's Note: MDPI stays neutral with regard to jurisdictional claims in published maps and institutional affiliations.

Copyright: © 2021 by the authors. Licensee MDPI, Basel, Switzerland. This article is an open access article distributed under the terms and conditions of the Creative Commons Attribution (CC BY) license (http://creativecommons.org/licenses/by/4.0/).

Table S1. Details of target definitions.

Target Volume	Definition	Note
Gross tumor volume (GTV)	MRI findings and gynecological examination immediately before each treatment planning.	
Clinical target volume 1 (CTV1)	Whole-pelvic irradiation included all areas of gross and potentially microscopic disease, which consisted of the primary site (GTV, whole uterus, parametrium, ovaries, and at least the upper half of the vagina) and the whole pelvic node region (enlarged lymph node, common iliac, internal iliac, external iliac, obturator, and presacral node regions).	
Planning target volume 1 (PTV1)	CTV1 plus a 10 mm safety margin for positioning uncertainty.	Consider an additional margin (+5mm) for uterine position uncertainty by checking several times of CT images.
Clinical target volume 2 (CTV2)	Uterus with GTV, parametrium, and swollen pelvic lymph nodes.	
Planning target volume 2 (PTV2)	CTV2 plus a 5 mm safety margin for positioning uncertainty.	Consider an additional margin (+5mm) for uterine position uncertainty by checking several times of CT images.
Clinical target volume 3 (CTV3)	Primary GTV	
Planning target volume 3 (PTV3)	CTV3 plus a 0–5 mm margin.	The gastrointestinal tract was excluded from the PTV3.

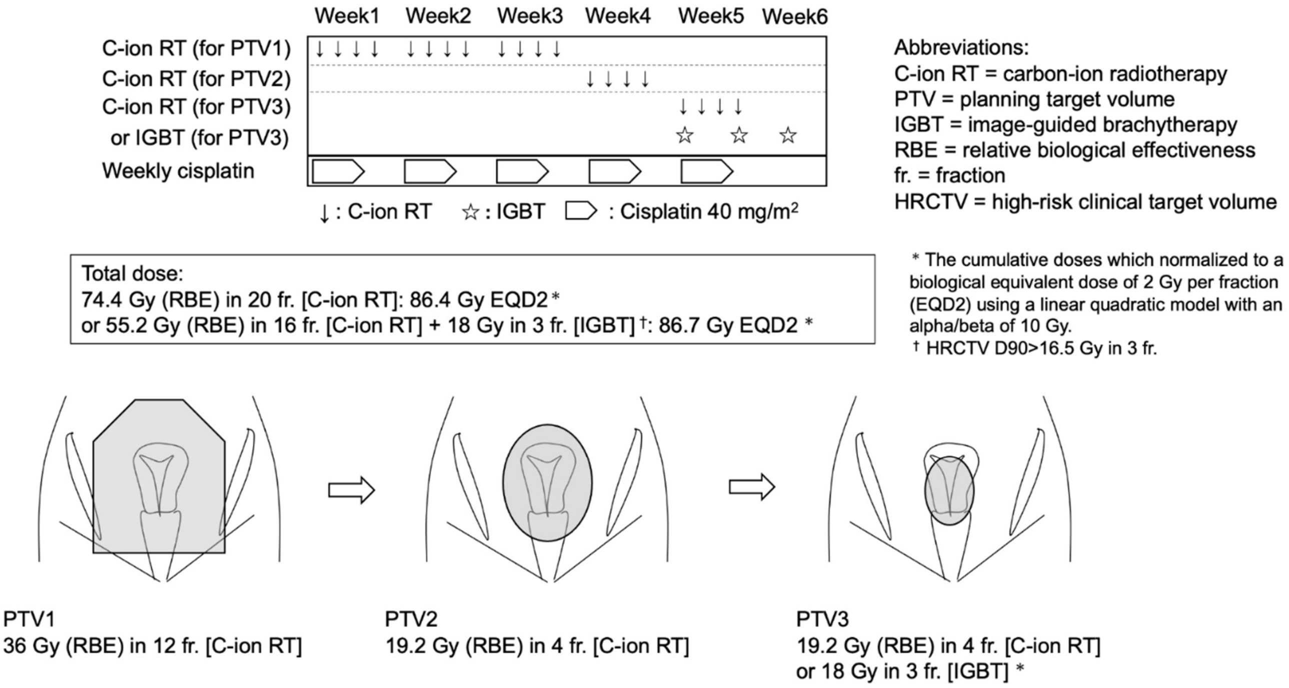

Figure S1. Outline of treatment of the present study.
